# Rotational Thromboelastometry-Guided Venoarterial Extracorporeal Membrane Oxygenation in the Treatment of Amniotic Fluid Embolism

**DOI:** 10.1155/2022/9658708

**Published:** 2022-05-18

**Authors:** Jillian K. Wothe, Elizabeth Elfstrand, Michael R. Mooney, Donald D. Wothe

**Affiliations:** ^1^University of Minnesota, Medical School, 420 Delaware St SE Minneapolis, MN, 55455, USA; ^2^Abbott Northwestern, Department of Obstetrics and Gynecology, 800 E 28th St Minneapolis, MN 55407, USA; ^3^Abbott Northwestern Department of Cardiology, 800 E 28th St Minneapolis, MN 55407, USA

## Abstract

Amniotic fluid embolism (AFE) is a rare and often fatal complication of pregnancy that occurs during the puerperium. The low incidence of AFE has resulted in few large studies, which makes evidence-based management of AFE challenging. The use of extracorporeal membrane oxygenation (ECMO) has been reported but is limited by availability and challenges managing anticoagulation. In this report, we detail the case of a 29-year-old female who suffered from an AFE leading to cardiac arrest and disseminated intravascular coagulopathy. She was treated with protocolized A-OK (adenosine, ondansetron, and ketorolac), emergency c-section, cardiopulmonary resuscitation, massive blood transfusion, and rotational thromboelastometry-guided ECMO, allowing her to forgo initial anticoagulation. After a prolonged rehabilitation with initial poor neurological status, she made a complete recovery. In this report, we describe the protocols that contributed to her recovery and detail management of complicated AFE for other clinicians.

## 1. Introduction

Amniotic fluid embolism (AFE) is a rare and often fatal complication of pregnancy that occurs during the puerperium [1]. It is a clinical diagnosis and one suggested set of diagnostic criteria consists of sudden onset of cardiac arrest or hypotension, overt disseminated intravascular coagulation (DIC), absence of fever, with onset during labor, or within 30 minutes of delivery of the placenta [2]. The low incidence of AFE (estimated 1 in 40,000 births) has resulted in few large studies so many hospitals have no evidence-based protocols [1]. Management focuses on cardiopulmonary resuscitation, delivery of the fetus, and correction of the coagulopathy [3]. Patients also suffering from hemorrhage are treated with massive blood transfusion (MBT) and tranexamic acid [4]. Some case reports have suggested a combination therapy of atropine, ondansetron, and ketorolac (A-OK) may improve survival; however, this treatment remains investigational [5]. The use of extracorporeal membrane oxygenation (ECMO) has been reported but its use is limited by availability and challenges managing anticoagulation [6–8]. In this report, we detail the case of a 29-year-old female who suffered from an AFE requiring cardiopulmonary resuscitation (CPR), MBT, rotational thromboelastometry- (ROTEM-) guided ECMO, and prolonged rehabilitation with initial poor neurological status ending in complete recovery.

## 2. Case

The patient is a 29-year-old gravida 1 para 0 female with no significant medical history who presented to our tertiary care hospital at 39+0 weeks for induction of labor due to gestational diabetes and patient request. Her pregnancy was otherwise normal, and she received routine prenatal care and screenings throughout the pregnancy.

She presented in the evening for cervical ripening and was started on vaginal Cytotec. Her blood pressure at the time of induction was 110/60, While her amniotic fluid levels were not assessed at the time of induction, they had been normal throughout her pregnancy. In the early morning, she had one episode of fetal tachysystole. This was treated with oxygen, 500 cc of fluids, and terbutaline. She continued laboring and at 7 : 34 AM, and she got up to go to the bathroom. At that time, fetal heart rate tracing showed a few late decelerations. She suddenly did not feel well and slumped forward at 7 : 49 AM. The staff assist button in the labor room alerted others, and she was quickly placed on her right side and given oxygen and her vital signs were assessed. Fetal heart rate was noted to be in the 60 s. She was quickly moved to the operating room at 7 : 54 AM. Several nurses, anesthesia staff, and obstetric physicians quickly assembled for an emergency cesarean section. The anesthesiologist anesthetized and intubated her while also administering the A-OK medications (atropine, ondansetron, and ketorolac). The fetus was delivered at 8 : 04 AM via emergency cesarean section with minimal bleeding, one minute after the general anesthetic was delivered. Placental pathology later showed that it was in the 75^th^ percentile for weight and had a three-vessel cord. There was focally increased perivillous fibrin deposition. The placenta was negative for chorioamnionitis and villitis, and 1% of the placental volume had infarct.

**Figure 1:**
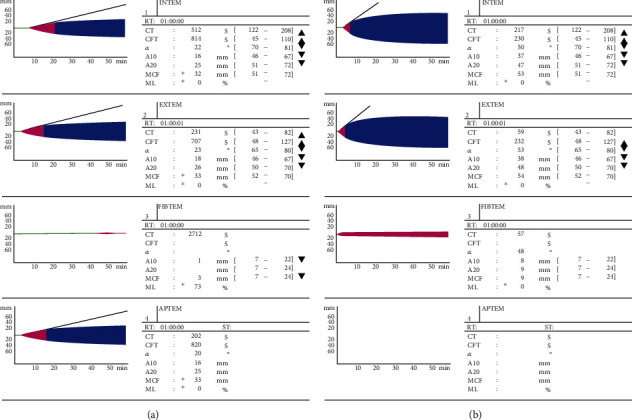
Initial and subsequent rotational thromboelastometry (ROTEM) parameters for a patient with amniotic fluid embolism. (a) ROTEM obtained just after ECMO initiation demonstrates extremely prolonged clotting time, poor clot amplitude, and fibrinolysis. (b) ROTEM obtained after additional FFP and cryoprecipitate showing dramatic improvement with normal EXTEM clotting time, mildly prolonged INTEM clotting time, normal FIBTEM clot amplitude, and EXTEM/INTEM clot amplitude just below the normal range.

Following delivery, there was a significant drop in maternal blood pressure without notable hemorrhage. The patient then had a pulseless electrical activity (PEA) arrest followed by pulseless ventricular tachycardia. CPR was started, and she was put on a Lucas CPR device with excellent end tidal carbon dioxide readings in the high 20-30 s. She was defibrillated multiple times and treated with epinephrine and amiodarone. During the compressions, she exhibited spontaneous movements. She was noted to have signs of DIC during blood draws and at the c-section incision site, so she was started on a massive transfusion protocol guided by ROTEM. She ultimately received nearly 7 liters of blood products including packed red blood cells, cryoprecipitate, fresh frozen plasma, and tranexamic acid. Point of care ultrasound showed dilated right ventricle with poor function and hyperdynamic, underfilled left ventricle with estimated ejection fraction of 20%. Due to her depressed cardiac function, the extracorporeal cardiopulmonary resuscitation (ECPR) team was called, and she was cannulated on peripheral venoarterial- (VA-) ECMO via right femoral artery and vein at 9 : 00 AM. She was not given heparin due to ROTEM with prolonged clotting time and very low clot amplitude with evidence of fibrinolysis (Figure 1). Initial ROTEM obtained just after ECMO initiation demonstrated extremely prolonged clotting time, poor clot amplitude, and fibrinolysis. ROTEM obtained after additional FFP and cryoprecipitate showing dramatic improvement with normal EXTEM clotting time, mildly prolonged INTEM clotting time, normal FIBTEM clot amplitude, and EXTEM/INTEM clot amplitude just below the normal range. She was started on cefepime and vancomycin prophylactically.

Postoperatively, she developed high-pressure cardiogenic pulmonary edema due to the MBT and increased afterload from VA-ECMO, so she was diuresed with Lasix and given nitroprusside to reduce afterload. At that time, her neurological exam was good as she moved her eyes and was able to follow commands. This was supported by excellent cerebral near infrared spectroscopy (NIRS). Electroencephalogram (EEG) did not detect seizure activity. She did have acute kidney injury, which improved with fluids. The day after her cardiac arrest, she developed leukocytosis and rigors, so metronidazole was added to her antibiotic regimen. Her DIC was resolved, and she was started on heparin. She remained on ECMO for two days, during which time she required minimal support and had excellent pulse pressures on dobutamine only. At the time of decannulation, her echocardiogram had improved to an EF of 60-65% with normal right heart function. Prior to decannulation, turndown to VA-ECMO at 1 liter per minute showed no change in blood pressure, right ventricular function, or left ventricular function.

Following decannulation, her sedation was weaned, and she no longer followed commands. She also developed stimulus induced myoclonus and left gaze deviation. Neurology was consulted and had concern for cefepime neurotoxicity, and this was discontinued along with sedation other than dexmedetomidine. She was started on levetiracetam for the myoclonus. Magnetic resonance imaging showed multiple bihemispheric strokes, changes consistent with posterior reversible encephalopathy syndrome (PRES), and a few areas with tiny hemorrhages; however, her cortical ribbon was intact. EEG showed severe slowing but no epileptiform activity. Neurology felt PRES was likely during the resuscitation given normotensive postoperative course. CT angiography was negative for reversible cerebral vasoconstriction syndrome and postpartum angiopathy. Over the next few days, she had slow improvements and began to move the left side spontaneously. Neurologic status was difficult to assess due to the continued need for sedation due to agitation. Twenty days after the initial event, she was weaned from the ventilator and her sedation was discontinued. At that, time her level of consciousness improved markedly, and she suddenly started following commands and mouthing answers to questions. An echocardiogram showed normal function. She was discharged to an acute rehab facility with right arm weakness, left gaze preference, dysphagia, dysarthria, and impaired balance and cognition. She was discharged to home within 6 days with no significant cognitive deficits. Her consent was obtained for this case report.

## 3. Discussion

**Figure 2:**
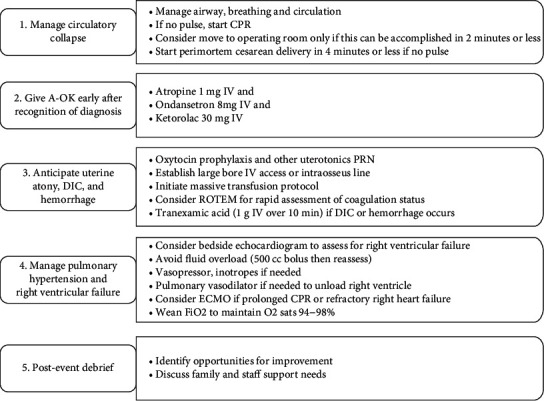
Institutional protocol for AFE which is based on the checklist published by the Society for Maternal Fetal Medicine.

This is a case of massive cardiovascular collapse due to AFE that was successfully treated with VA-ECMO with ROTEM and protocolized treatments, including A-OK, MTP, and a code system which together helped save the patient's life. Our protocol, which is adapted from the checklist published by the Society for Maternal Fetal Medicine, is shown in Figure 2 [9]. This case is unique due to the use of ROTEM-guided ECMO, which is an uncommon strategy for managing AFE that likely contributed to our patient's survival.

AFE is a clinical diagnosis of exclusion. Our patient presented classically with a preceding aura. She was felt to have AFE due to acute cardiopulmonary failure, evidence of right heart strain on echocardiogram, lack of fever, clinical onset during labor, overt DIC with platelets less than 100, INR greater than 2.5, and fibrinogen less than 200. Ultrasound for deep vein thrombosis was negative, and electrocardiogram was without ischemic changes, making myocardial infarction and pulmonary embolism unlikely. This meets the clinical criteria proposed by Clark et al. in 2016 [2]. AFE has a risk of mortality of 86% and can result in both maternal and neonatal death if delivery is not expedited, and preparations were made for the inevitable sequela. It presents an excellent opportunity to do simulation to prepare for this rare and often fatal complication.

In this case, prompt nursing response to move the patient quickly and involve the entire team and rapid recognition of the diagnosis with appropriate treatment of AFE was critical to the patient's survival. We believe our patient survived because of a rapid diagnosis and use of these protocolized interventions. At our institution, we have massive transfusion protocols specific to obstetric patients and predosed A-OK therapy in the crash carts. We also have a system called “Code OB” which allows rapid response to catastrophic obstetric complications. Finally, our institution is fortunate to have a hospital wide ECMO-CPR team that was able to respond promptly when the patient suffered cardiovascular collapse.

The use of ECMO in patients with AFE is uncommon, since many hospitals lack the capability and because of the coagulopathies inherent to AFE. In this case, multiple rounds of CPR and severe cardiac dysfunction diagnosed by echocardiogram necessitated ECMO support. To account for the underlying coagulopathy, ROTEM was used to calibrate anticoagulation. As a result, our patient was not initially anticoagulated with heparin. Balancing anticoagulation has previously been a major barrier to using ECMO in patients with AFE, as they often have severe underlying coagulopathies that make ECMO dangerous [6]. More recently, changes have been made to the ECMO circuits and oxygenators that make the use of ECMO without anticoagulation feasible [10]. These include coating the circuits with heparin and reduction of contact with foreign material in the circuit [10]. As a result, multiple studies have demonstrated successful use of ECMO in populations that were previously ineligible [11]. While no large studies have been done on using ECMO in the treatment of AFE, recent case reports have demonstrated its successful use [7, 8].

In conclusion, amniotic fluid embolism is a catastrophic complication of pregnancy that occurs during the peripartum period. Our case emphasizes that with prompt recognition followed by the use of protocolized treatments and rotational thromboelastometry-guided VA-ECMO in patients suffering massive cardiovascular collapse due to AFE, survival is possible even in the most critically ill patient.

## Data Availability

Data is available upon request by emailing wothe003@umn.edu.
